# A Review of Nonhuman Primate Models of Rift Valley Fever Virus Infection: Progress, Challenge Strains, and Future Directions

**DOI:** 10.3390/pathogens13100856

**Published:** 2024-10-01

**Authors:** Kimimuepigha Ebisine, Darcy Quist, Stephen Findlay-Wilson, Emma Kennedy, Stuart Dowall

**Affiliations:** UK Health Security Agency, Porton Down, Salisbury SP4 0JG, UK; kimi.ebisine@ukhsa.gov.uk (K.E.); darcy.quist@ukhsa.gov.uk (D.Q.); stephen.findlay-wilson@ukhsa.gov.uk (S.F.-W.); emma.kennedy@ukhsa.gov.uk (E.K.)

**Keywords:** rift valley fever, nonhuman primate, vaccine, model

## Abstract

Rift Valley fever (RVF) is a mosquito-borne viral disease that primarily affects animals, especially ruminants, but has the capacity to infect humans and result in outbreaks. Infection with the causative agent, RVF virus (RVFV), causes severe disease in domestic animals, especially sheep, resulting in fever, anorexia, immobility, abortion, and high morbidity and mortality rates in neonate animals. Humans become infected through exposure to infected animals and, less frequently, directly via a mosquito bite. A greater awareness of RVFV and its epidemic potential has resulted in increased investment in the development of interventions, especially vaccines. There is currently no substitute for the use of animal models in order to evaluate these vaccines. As outbreaks of RVF disease are difficult to predict or model, conducting Phase III clinical trials will likely not be feasible. Therefore, representative animal model systems are essential for establishing efficacy data to support licensure. Nonhuman primate (NHP) species are often chosen due to their closeness to humans, reflecting similar susceptibility and disease kinetics. This review covers the use of NHP models in RVFV research, with much of the work having been conducted in rhesus macaques and common marmosets. The future direction of RVF work conducted in NHP is discussed in anticipation of the importance of it being a key element in the development and approval of a human vaccine.

## 1. Introduction

Rift Valley fever virus (RVFV), recently renamed Phlebovirus riftense, belongs to the *Phenuviridae* family within the genus Phlebovirus, alongside multiple other viruses grouped based on their structural similarities [1]. The genome consists of three single-stranded RNA segments—large (L, 6.5–8.5 knt), medium (M, 3.2–4.3 knt), and small (S, 1.7–1.9 knt)—alongside an “ambisense” replication strategy [2,3,4]. Like most other phleboviruses, RVF is a vector-borne viral disease affecting both ruminants and humans.

### 1.1. Epidemiology

Rift Valley fever (RVF) was first discovered in 1930 on a farm situated near Lake Naivasha in the Rift Valley of Kenya when a high level of mortality was observed in newly born lambs [5]. The outbreak quickly spread to thousands of sheep on the farm with increased mortality and abortion among adult sheep with signs of high fever, thick mucopurulent nasal discharge, vomiting, the passing of bloody stools in some cases, and refusal to feed [5]. Several other outbreaks of RVF have been reported across many African countries, including Egypt, South Africa, Madagascar, Uganda, Mauritania, Senegal, and the Gambia [6,7,8,9,10]. Whilst the most prone species to RVF are domesticated ruminants such as sheep, goats, cattle, camels, and domestic pigs, serological evidence shows infection in several other wild animals such as warthogs, impalas, giraffes, and humans [11,12,13,14,15,16]. The unique climatic and environmental conditions have been cited as facilitating conditions for the spread of the virus in the regions, including heavy rainfall, periodic flooding, increased mosquito population, unvaccinated susceptible livestock, and a high level of livestock trade [9,17]. 

The first observed RVF cases in Egypt were recorded in 1977, causing over 600 deaths and high economic losses in livestock and humans, outbreaks reoccurred in 1993, 1994, 1997, and 2003, affecting several regions of the country [18,19,20,21]. The disease was first recorded outside of Africa in the year 2000 when it was observed in Saudia Arabia and the adjoining Yemeni territories affecting over 40,000 animals and 883 humans (including 124 fatalities) in Saudi Arabia and 1328 cases (including 166 fatalities) in Yemen [22,23]. RVFV has since been identified in over 30 countries, including Turkey [24]. Studies carried out in countries surrounding the Mediterranean, including Turkey, Tunisia, Iran, Iraq, and Algeria, reported the presence of RVF in animals and humans [25,26,27,28,29]. A recent report in 2023 reported an outbreak of RVF in Uganda causing eight deaths [30].

Over the years, several cases of human infection with RVFV have been documented. Infections in laboratory workers were documented during the outbreak in Kenya, South Africa, and Egypt [5,31,32,33,34]. Immunological studies in humans indicate a seroprevalence range of RVFV exposure of 1.8% in Kenya [35], 11.1% in Saudia Arabia [36], 1.4–1.9% in Tunisia [25,37], 4.9% in Turkey [38], and 10% in Spain [39]. 

Although no outbreak of RVF has been reported in Europe, the risk of its introduction to other continents, including Europe, is high due to its wide range of hosts and the global distribution of arthropod vectors in Italy and Europe [39,40]. The UK Health Security Agency (UKHSA), in collaboration with the Animal and Plant Health Agency (APHA) and others, has demonstrated that UK mosquito species possess transmission potential for RVFV in the laboratory setting, albeit at a low efficiency [41].

### 1.2. RVF Disease and Vaccination

The main route of RVFV infection in humans is through direct contact with infected livestock and tissues, blood, or fluids from infected animals, although aerosols have also been implicated by laboratory workers and field professionals [21,42]. Human infection can also occur directly via bites from infected mosquitoes; however, the symptoms are often mild with recovery occurring without major consequences. Human RVF disease is characterised by abrupt onset of fever, chills, and general malaise after an incubation period of two to six days. Some severe cases have been reported to affect approximately 1–2% of infected individuals and are characterised by acute-onset liver disease, delayed-onset encephalitis, retinitis, blindness, or a hemorrhagic syndrome, with a 10% to 20% rate of hospitalisation and reported cases of deaths [5,17,43,44,45].

Approximately 100 years after the first recorded outbreak, RVF still presents a severe economic impact on humans and livestock, causing tens of thousands of human cases resulting in hundreds of fatalities alongside over 100,000 deaths in domestic animals [46]. The unavailability of an approved vaccine for human use, despite there being several vaccines for livestock, has resulted in RVFV being considered a priority pathogen by the World Health Organization (WHO) R&D Blueprint [47], UK Vaccine Network (UKVN) [48], and Coalition for Epidemic Preparedness Innovations (CEPI) [49], resulting in the financing of two human vaccines with one already in clinical trials [50,51]. The current international response to RVFV combines collaboration, coordination, and communications between communities, physicians, and veterinarians in a One Health approach to contain and prevent outbreaks [30,40].

To effectively demonstrate efficacy, traditional human Phase III clinical trials may not be feasible for the development of vaccines against outbreak diseases such as RVF where occurrence is intermittent [51]. Alternative regulatory approval pathways exist, including evaluation through the Animal Rule pathway [52,53]. This requires a thorough investigation of various disease models, with nonhuman primate (NHP) species often being optimal for recapitulating human disease due to their comparable physiology to humans. In preparation for the advancement of RVF vaccines through the Animal Rule pathway, this review provides a detailed analysis of NHP models of RVF disease, examining the infection route, virus strain, and pathological outcomes.

## 2. Nonhuman Primate (NHP) Disease Models

To replicate the natural infection of RVF in experimental conditions, animal models of RVFV infection are required. Several laboratory animal species have been shown to be susceptible, including mice [54], rats [55], hamsters [56], and ferrets [57,58]. These models have been the subject of other reviews [59,60]; therefore, the focus of this article is on NHP models of RVFV infection.

NHPs are classified into Old World and New World based on geographical location. The order primates, and specifically the infraorder Simiiformes (simians), which include the primates relevant for RVFV research, are subdivided into Catarrhini (apes and Old World monkeys) and Platyrrhini (New World monkeys) [61]. Old World monkeys are a large family native to Africa, Asia, and Europe; they include macaques and green monkeys [62]. The New World monkeys are a smaller family, which includes marmosets and squirrel monkeys distributed across tropical regions of the Americas. Although Old World monkeys are closer phylogenetically to humans, New World monkeys are often smaller and cheaper to house/feed, whilst still having a comparable immunological repertoire to humans [63]. Together, both Old and New World monkeys have been used as models in RVFV research.

### 2.1. Old World Monkeys

#### 2.1.1. Rhesus Macaques (*Macaca mulatta*)

The first studies of the RVFV challenge in NHP were reported in 1931, the same year as RVFV was identified, in a brief article reporting that rhesus macaques were susceptible after inoculation with sheep and human blood resulting in a fever lasting 2–3 days, with a full account published shortly after [64]. This subsequent full article comprehensively covered a range of details on RVFV, including the susceptibility of a range of animal species, and detailed the outcome of 14 rhesus macaques challenged with different inoculum and routes, including intraperitoneal (i.p.), intracerebral (i.c.) and intranasal (i.n.) [65]. Challenged NHPs developed viremia, assessed in those times via inoculating blood from the monkeys into mice and assessing survival, and fever in the majority of animals. The authors concluded that no fatal cases occurred in monkeys and that the fever associated with blood changes was similar to that recorded in humans [65].

The production of a neurotropic strain of RVFV, acquired through the intracerebral passage of brain homogenate at least 30 times in mice, was reported in 1936 [66]. Mouse brain homogenates from the passaging studies were inoculated into 10 rhesus macaques with different passage material, volumes, and routes consisting of i.p., i.c., and i.n. [66]. Seven challenged monkeys met moribund conditions, including encephalitis, and were subsequently culled, demonstrating that this virus strain was not only increasingly neurotropic for mice but also for other species, including monkeys.

Rhesus macaques were further utilised in the isolation of RVFV from wild-caught mosquitoes from the uninhabited Semliki Forest in western Uganda sampled in 1944 [67]. This study used six monkeys, alongside mice, to establish the infectivity of mosquito homogenate preparations and characterise subsequent virus preparations. These results provided direct evidence of the proof of transmission of RVFV by mosquitoes.

In 1962, a report on the aerosolisation of RVFV was generated from the US Army Biological Laboratories [68]. Sixteen monkeys were put into four groups of *n* = 4 and each challenged with different inhaled doses of RVFV, alongside a group of *n* = 4 control animals. All animals showed viremia with some animals having temperature elevations, demonstrating the respiratory route as a non-vector strategy for viral entry and subsequent disease progression.

An extensive review article of RVFV published in 1965 included highlights of work conducted as part of the author’s PhD thesis from 1961, including studies in rhesus macaques [69]. Scant information on specifics of the NHP work was included in the manuscript, but the summarised findings confirmed earlier reports of viremia post-challenge and febrile responses.

After a period of over 30 years, an extensive study of the RVF NHP model was described in 1986 by a team from the US Army Medical Research Institute of Infectious Disease (USAMRIID) where the effects of several drugs suspected of being active against other RNA viruses were tested for the efficacy of experimentally induced RVFV, with all four control animals developing viremia with no associated clinical illness [70]. For these studies, the challenge virus was the Zagazig Hospital 501 (ZH-501) strain of RVFV that had been passaged twice in diploid fetal rhesus lung cells (DBS-103) followed by an additional one in spleen and one serum passage in rhesus monkeys, thus showing some ‘adaptation’ in the NHP host. 

Subsequently, a further study by the by the same group at USAMRIID a couple of years later expanded the work to investigate the suitability of rhesus macaques as a model for human infection, detailing four experiments [71]. In the first study, animals were challenged subcutaneously (s.c.), and a comparison was made between infectious serum initially inoculated into diploid fetal rhesus lung cells (DBS-FRhL-2), and the virus from that at passage 1 (FRhL_1_) was compared with passage 2 (FRhL_2_). Only three animals were assessed, and out of two monkeys inoculated with the FRhL_1_ virus only one exhibited viremia on day 1, whilst a third monkey inoculated with the FRhL_2_ virus also developed viremia [71]. Despite the small sample size, it is noteworthy that two out of three monkeys were viremic, with no clear advantage observed for the lower passaged virus. In a second study, macaques were inoculated via the intravenous (i.v.) route, following on from unpublished observations at the time showing that this route was more sensitive in mice. Nearly all animals developed transient viremia with no clinical signs, with one macaque that presented with hemorrhagic diathesis and was euthanised on day 7. In the third experiment, a comparison between FRhL_2_-cultured virus was conducted against spleen homogenate-derived virus from the macaque that was culled in the second experiment due to the demonstration of severe disease. Group sizes of *n* = 3 were used, with viremia detected in all challenged animals; in one animal inoculated with the spleen-derived virus, haemorrhagic symptoms were exhibited but subsequently resolved. In the fourth experiment, five monkeys were challenged with a 1:100 dilution of the peak viremic sera from the animal meeting the endpoint criteria from the second study. All were viremic, with one showing clinical signs and subsequently euthanised on day 3. In summary, across the different studies, approximately 20% of challenged macaques developed haemorrhagic fever, which was associated with extensive liver necrosis, disseminated intravascular coagulation, and microangiopathic hemolytic anemia in severely affected animals, documenting the first examples of severe RVF disease in NHPs.

In 1989, the USAMRIID team further studied the effect of interferon (IFN)-α given either as prophylaxis or therapeutically, showing protective effects of the treatment [72]. The study design consisted of six groups each of 4–5 animals receiving IFN-α alongside a control group totalling 17 monkeys receiving sterile diluent. All control animals intravenously challenged with RVFV developed viremia beginning at 24 h and peaking by 48 h post-challenge. Three animals developed severe clinical signs including anorexia, cutaneous haemorrhage, epistaxis, vomiting, and death. Of the remaining 14 animals, 7 demonstrated clinical illness characterised by cutaneous rash and vomiting or anorexia. Body temperatures were monitored in a cohort of 11 animals, with all experiencing a febrile response that paralleled viremia. These 17 animals that served as control animals for the series of four experiments investigating the efficacy of IFN-α were additionally used to study the role of the IFN response during infection [73] and haematological changes [74]. Furthermore, 12 of these animals were the subject of a separate manuscript on the kinetics of serum viral antigen and antibody responses [75].

To follow-up on the protective responses observed with IFN-α, studies were expanded to assess IFN-γ, with similar results demonstrated although no synergism was observed between the two IFN preparations when given prophylactically with combined low doses [76]. Untreated control rhesus macaques challenged intravenously with RVFV developed high-titre viremia, fever, and cutaneous petechiae. Notably, all monkeys in this study survived the virulent virus challenge; however, one animal developed cutaneous petechiae in the axillary and inguinal regions 48 h post-infection, which remained enlarged until day 6 post-infection and then began to resolve, becoming inconspicuous by day 14.

A comparative study on two strains of RVFV using rhesus macaques was reported in 2003: the mutagen-attenuated ZH-548 strain of RVFV (RVF ZH-548-P12), which led to the creation of the RVF MP-12 vaccine, and the virulent ZH-501 strain of RVFV in NHP [77]. Animals were intravenously inoculated with either the ZH-548-P12 or ZH-501 RVFV strain and monitored for 30 days. The study reported that all monkeys survived the intravenous inoculation with both virus strains, with those inoculated with the ZH-548-P12-attenuated vaccine strain remaining clinically well, with low viremia detected only at 24 h post-inoculation. In contrast, three out of four macaques inoculated with the ZH-501 virus strain developed anorexia, mild-to-moderate petechiae in the axillary and inguinal regions, reduced activity 3–5 days post-inoculation, and detectable viremia for 3–5 days post-inoculation. 

In 2011, two more reports on the RVF MP-12 vaccine performed in rhesus macaques were published. The first was on the investigation of immunogenicity and efficacy after mucosal immunisation delivered via either aerosol exposure or oral administration [78]. Group sizes of *n* = 4/group were used due to space limitations and minimising the use of animal numbers whilst still demonstrating proof-of-concept data. All aerosol-immunised animals and 2/4 orally immunised animals developed neutralising antibodies that protected against clinical signs and viremia after an aerosol challenge with virulent RVFV. In contrast, unimmunised control animals and the remaining 2/4 orally immunised animals that did not seroconvert developed viremia and elevations in rectal temperatures. A second study intramuscularly immunised animals and assessed vaccine efficacy via parenteral and aerosol routes of challenge [79]. After the intravenous challenge, vaccinated monkeys showed protection against viremia and clinical disease, whereas unvaccinated control animals exhibited signs of mild clinical disease and developed high-titre viremia. For vaccinated animals that were challenged via small particle aerosol exposure, there was no evidence of clinical disease and no viremia or oral virus detectable. This is in contrast to unvaccinated control animals where a short duration of viremia was observed alongside viral isolations from oropharyngeal swabs.

In 2011, USAMRIID researchers published an article evaluating common marmosets as an alternative NHP model for RVF disease [80]. For comparison, four rhesus macaques were intravenously challenged with RVFV. Viremia developed in all subjects, peaking on day 2 post-challenge, but none succumbed to or presented with clinical illness. One rhesus macaque experienced a slight increase in temperature, peaking on day 6 post-inoculation. A similar study was conducted in 2014 where researchers at the University of Pittsburgh assessed four NHP species for susceptibility to RVFV via aerosol exposure: cynomolgus macaques, rhesus macaques, African green monkeys, and common marmosets [81]. In the study involving two rhesus macaques, a biphasic fever was observed, with no other clinical signs indicative of disease present, and neither of the subjects succumbed to the infection. Nonetheless, they displayed a persistent elevation in body temperature for 30 days following infection [81].

Results from RVFV infection studies conducted in rhesus macaques have been summarised in Table 1.

#### 2.1.2. Cynomolgus Macaques (*Macaca fascicularis*)

The first report of RVFV challenge of a cynomolgus macaque was a single animal intracerebrally inoculated with mouse brain homogenate in 1936 [66]. Given the route of the challenge and the addition of starch to the inoculum, it was unsurprising that the animal developed encephalitis and was culled 6 days after the challenge.

In 1965, referencing work undertaken in their PhD thesis, Bernard Easterday indicated that no evidence existed for differences between RVFV susceptibility in cynomolgus macaques and rhesus macaques [69]. Unfortunately, access to the source document was not possible during the preparation of this review article to provide further details. Of note, in these early publications, the species is referred to as *M. irus*, prior to *M. fascicularis* being widely used for ‘crab-eating macaques’, now widely named cynomolgus macaques in the literature.

More recently, in 2014, two cynomolgus macaques were used to assess susceptibility to aerosol exposure of RVFV alongside other NHP species [81]. Similar to findings in the two rhesus macaques also tested, a biphasic fever was reported, but no other clinical signs indicative of disease were noted. Serial sampling was not conducted during these studies, so viremia levels were not addressed.

Results from RVFV infection studies conducted in cynomolgus macaques are summarised in Table 2.

#### 2.1.3. Other Old World Species

In addition to the laboratory macaque species, other Old World primate species have been reported for their susceptibility to RVFV infection.

In an article published in 1932, two animals of each of the three African monkey species were tested for their infectivity to RVFV: the green guenon (*Cercopithecus callitrichus*), the sooty mangabey (*Cercocebus fuliginosus*), and the Patas guenon (*Erythrocebus patas*) [83]. Temperature rises were observed in a single green guenon and a single Patas guenon animal. Blood from all six animals was collected and inoculated into mice to assess infectivity, with viremia being ascertained in all of the challenged monkeys.

Baboons were tested for RVFV susceptibility in a study reported in 1972 where four adolescent animals were inoculated with the van Wyk strain, and two animals were kept as a negative control [84]. Viremia was present in all challenged baboons, persisting for 3–4 days and correlating with raised body temperature; however, no overt clinical disease signs were produced.

In 2014, a group of six African green monkeys (*Chlorocebus aethiops*) were included in a study that was challenged via the aerosol route with 4.90–5.86 log_10_ pfu RVFV [81]. All developed fever, with five of the six animals succumbing to the disease by day 10 to 11 post-challenge. Clinical signs of those who developed severe disease included dehydration and anorexia, alongside evidence of neurological disease, particularly excessive drooling.

### 2.2. New World Monkeys

#### 2.2.1. Common Marmosets (*Callithrix jacchus*)

Marmosets were first reported for RVFV susceptibility in 1932 [83]. Three animals were subcutaneously inoculated with blood from RVFV-infected mice, two *Hapale (=Callithrix) jacchus* and one *Hapale (=Callithrix) penicillata*. After the challenge, a short febrile period was followed by subnormal temperature drops. The two common marmosets recovered, but the *Hapale penicillata* died seven days after inoculation although the cause was not conclusive as RVFV due to only slight necrosis of the liver cells and the presence of pneumococcus in this organ.

In 2012, a larger study on RVFV in marmosets was published [80]. Twenty healthy adult marmosets were split into four challenge groups via three exposure routes: i.v., s.c. (two doses), and i.n., with the remaining group of animals used as negative controls. All test animals developed viremia. Among the marmosets exposed via the i.v. route, one succumbed on day 2 post-infection, while two others exhibited clinical symptoms such as anorexia, reduced activity, and ruffled fur with a hunched posture. The study noted that the marmoset infected intravenously and deceased on day 2 post-infection had fibrin deposition and thrombi in various tissues, indicating disseminated intravascular coagulation at death. Of the marmosets exposed via the s.c. route, two succumbed or were euthanised, and three out of four exhibited clinical symptoms similar to those exposed via the i.v. route. Marmosets inoculated with 7log_10_ pfu RVFV subcutaneously saw one animal perish on day 4 post-inoculation and another on day 11. When given 5log_10_ pfu subcutaneously, one marmoset was euthanised on day 7 and another on day 12 due to neurological impairments. A 100% mortality rate occurred in marmosets inoculated intranasally, meeting endpoints on days 8–11 and exhibiting neurological impairments.

In 2014, a comparative study of NHP models aimed to determine whether a lethal disease could be induced in NHPs through aerosol infection with RVFV, including in common marmosets [81]. Eight marmosets exposed to increasing doses of aerosolised RVFV, from 1.78 to 5.18 log_10_ pfu, developed a biphasic fever, peaking initially between days 3 and 6 and again from day 7 to day 15 post-exposure. Four marmosets succumbed to the disease and were euthanised between days 9 and 10 post-infection. These marmosets also exhibited clinical signs including anorexia, dehydration, seizures, and instability.

Seventeen marmosets were used in a study to assess the immunogenicity and efficacy of recombinant RVFV with complete deletions in the NSs and NSm genes as vaccine candidates [85]. A group of five sham-vaccinated controls were challenged via the aerosol route alongside the vaccinated animals with 6.4 log_10_ pfu RVFV. In contrast to earlier studies [81], none of the challenged animals exhibited clinical signs of illness or experienced significant weight loss or temperature changes, with all surviving to day 28 post-challenge study end date. Viremia was detected during the course of the study and control animals had detectable viral RNA in multiple tissues collected at necropsy.

In testing live-attenuated vaccine approaches, the marmoset model was again employed for immunogenicity and efficacy testing [86]. Two experiments were reported, with the first using the wild-type RVFV 35/74 for the challenge. Of four control animals, one showed tremors starting on day 4 post-inoculation and worsened on day 15 when the animal was euthanised. Of note, this animal also presented with substantial weight loss and had unaccountable weight loss prior to inoculation, so it may have had an underlying condition. The remaining three animals showed no clinical signs. In the second study, animals were challenged with the wild-type strain 35/74. Four of six control animals developed clinical signs, including unkempt coats and hunched postures, with two improving and two meeting humane endpoints on days 13 and 14 post-challenge. All RVFV-challenged marmosets across the two studies had pronounced temperature increases and viremia 2–4 days post-challenge.

Results from RVFV infection studies conducted in common marmosets are summarised in Table 3.

#### 2.2.2. Other New World Species

In the first report of the marmoset RVFV challenge published in 1932, three capuchin monkeys were also compared: two *Cebus fatuellus* and one *Cebus chrysopus* [83]. All showed a rise in temperature and viremia, with blood removed demonstrating infectivity for mice.

In 1965, two spider monkeys (*Ateles ater*) were reported to be refractory to RVFV infection compared with rhesus and cynomolgus macaques [69].

## 3. Virus Challenge Strain

To allow comparison between studies, alongside a pathway of using data generated in NHP to advance medical countermeasure licensure, the choice and standardisation of RVFV challenge strain plays a critical role. In the US Food and Drug Administration (FDA) guidelines on product development under the Animal Rule, the challenge agent should be (i) the same as the etiological agent that causes the human disease and is associated with outbreaks of disease; (ii) based on known virulence factors; (iii) be of a low passage history; and (iv) characterised, for example, passage history, method of preparation, and concentration [87].

With another virus that causes haemorrhagic fever, the Ebola virus (EBOV), well-characterised challenge stocks have gained support across the filovirus community [88]. Similar discussions have also been undertaken with the Venezuelan equine encephalitis virus (VEEV) where efficacy testing of vaccines under the Animal Rule is necessitated [89]. For VEEV challenge stocks, viruses originating from three populations were considered for their advantages and disadvantages: uncloned (wild-type), plaque-cloned, and cDNA-cloned, with the recombinant approach being the preferred option [89]. For EBOV, the approach of generating a cloned virus has been reported [90] but was not considered a feasible approach for generating standardised challenge material due to lot-to-lot variability being observed, including different consensus sequences between batches [89]. When a mouse-adapted wild-type EBOV was compared with a recombinant virus, the kinetics of the viral infection were different with the cloned virus having higher and earlier viral loads in the blood and tissues of challenged mice [91]. The use of cloned viruses is further complicated due to genetic modification regulations, which vary between countries, biodefence implications, and restrictions where the methodology has been protected by patents, e.g., recombinant RVFV [patent No. US8673629B2].

For RVFV, the most widely used strain for the challenge of NHPs is ZH501, except for one study in marmosets (Table 4). In this latter aforementioned study, two wild-type strains were used in control groups for different experiments (74HB59 and 35/74), with disease severity being most pronounced in 74HB59 [86]. Therefore, RVFV strain differences have been observed in the NHP model.

A pairwise comparison of the L, M, and S segments of the challenge strains of RVF used in NHP studies revealed a genetic dissimilarity ranging from 2 to 5.1%, with the most pronounced variation occurring within the M segment, although a comparatively high dissimilarity was also observed within the NSs region of the S segment, considering its size of ~798 bp (Table 5) [93].

The nonstructural protein NSs is the main virulence factor for RVFV, blocking transcriptional upregulation of antiviral type I interferons (IFN) and disrupting the general transcription factor TFIIH subunit p62 via the ubiquitin/proteasome pathway [94]. Studies in mice have shown that mutations in the NSs region result in differences in the clinical, pathological, and host–gene expression outcome, although other results suggest that functionality is more dependent on the conformational integrity of the NSs protein [95,96,97]. The nucleotide disparities in NSs between the different challenge strains of RVFV reported translate to nine different amino acid changes within this protein (Table 6). Although none of the specific amino acid changes have been assessed for virulence, an MP-12 strain of RVFV containing an M250K mutation was shown to have reduced virulence in CD-1 mice [93].

The culture of RVFV is also an important consideration. When developing a mouse model of RVF disease, we previously demonstrated that the virus grown in a mosquito cell line (C6/36) resulted in a more rapid disease progression than that grown in mammalian cells (Vero E6) although the lowest dose causing uniform severe disease was the same for both preparations [54].

## 4. Discussion and Future Direction

As described herein, the majority of work conducted with RVFV in NHP has been with rhesus macaques and common marmosets. Due to their outbred nature and use of small group sizes, differences have been observed in both species between similar experiments with the same challenge routes, particularly in the onset of clinical signs. Whilst marmosets generally, but not consistently, show increased clinical signs after the RVFV challenge, they confer several disadvantages, including limited blood volume withdrawal and increased evolutionary distance from humans compared with rhesus macaques [63]. Due to viremia being the most consistent readout of RVFV infection studies, restrictions on blood sampling will likely hamper study designs. In addition, for the studies of vaccines, immune monitoring from blood samples also provides a wealth of important data on immunogenicity responses. Macaques and marmosets have both been shown to be susceptible to filovirus infection [98], similar to RVFV in the high likelihood of requiring a nontraditional regulatory pathway for vaccine approval. Interestingly, the US Biomedical Advanced Research and Development Authority (BARDA) has supported two natural history studies with the Marburg virus and Sudan virus in preparation for use in the FDA Animal Rule approach using cynomolgus macaques [99], implying preference of this macaque species over marmosets. A recent report on the immunogenicity of RVF mRNA vaccines used mice and rhesus macaques, with the latter utilised due to being a model for human RVF disease [82], indicating an approach applicable in future testing of protective effects of this vaccine in this species. For RVFV, very few studies in cynomolgus macaques have been reported, indicating that further work in this species is required to ascertain susceptibility.

The use of viral challenge stocks also needs consideration in RVFV NHP studies, in particular the source of virus material. Whilst VEEV researchers have communicated with the FDA and gained concurrence on the use of cDNA-cloned methodology for the generation of viral stocks for efficacy studies, this was on the proviso that virulence was demonstrated to be comparable or non-inferior to wild-type stocks [89]. In a rat model, recombinant RVFV has been demonstrated to give a mean time to death identical to those receiving wild-type virus [100]. As wild-type RVFV strains are readily accessible, we suggest that ZH501 is a good candidate for the prototype strain used allowing comparisons between studies to be easier ascertained. For wild-type stocks, full characterisation is essential, especially sequencing due to RVFV ZH501 having been reported to have a quasispecies nature [101].

Along with the strain of virus, dose, and route of infection, the susceptibility of the animal model to RVFV infection depends on the species of the animal used. However, variation in susceptibility to RVF within the same species has not been addressed. In the US, a targeted sequencing of 1845 rhesus macaques for genes linked to inherited human retinal and neurodevelopmental disease led to the identification of substantial nucleotide variants that may aid the refinement of rhesus disease models [102]. Therefore, there is a need to consider genotyping the targeted NHP species during the model development.

For filoviruses, the development of coordinated activities and sharing of information, including on NHP models and challenge virus parameters, has been accelerated through the Filovirus Animal Non-Clinical Group (FANG) [103]. The WHO assembled an animal model working group for SARS-CoV-2 [104], with expansion to other priority pathogens being implemented in the post-pandemic era. These and similar initiatives, including representation from regulatory agencies, will continue the active collaboration, and sharing of knowledge and pathways to countermeasure licensure are crucial to mitigating the threat from RVFV.

## Figures and Tables

**Table 1:** Overview of RVFV studies reported in rhesus macaques.

Publication	Challenge Dose	Challenge Route ^1^	Number ^2^	Outcomes
Findlay et al., 1931 [64]	Unknown	Unknown	Unknown	Fever and leucopenia.
Findlay, 1932 [65]	Unknown	i.p.	10	Viremia and fever.
		i.c.	1	Viremia and fever.
		s.c.	1	Viremia.
		i.n.	2	Viremia and fever.
Findlay et al., 1936 [66]	Unknown	i.c.	3	Viremia and encephalitis, resulting in being culled.
		i.p.	5	Viremia and encephalitis in 2 animals ^4^.
		i.n.	2	Viremia, fever and encephalitis.
Smithburn et al., 1948 [67]	Unknown	s.c.	6	Viremia and fever, dependent on mosquito homogenate inoculum.
Miller et al., 1963 [68]	2820 MIPLD_50_^5^	Aerosol	4	Viremia and temperature elevation.
	275 MIPLD_50_	Aerosol	4	Viremia and temperature elevation.
	145 MIPLD_50_	Aerosol	4	Viremia and temperature elevation.
	76 MIPLD_50_	Aerosol	4	Viremia and temperature elevation.
Easterday, 1965 [69]	Unknown	Unknown	Unknown	Viremia and febrile.
Peters et al., 1986 [70]	4.2 log_10_ pfu	i.v.	4	Non-clinically ill. All viremic for 3 days.
Peters et al., 1988 [71]	5.3 log_10_ pfu	s.c.	3	One animal non-viremic, two animals viremic (days 1–3).
	4.7 log_10_ pfu	i.v.	4	Three animals showed transient viremia; one ill with haemorrhagic diathesis and culled on day 7.
	4.1 log_10_ pfu	i.v.	3	All were viremic, with one ill with haemorrhagic signs but recovered.
	4.8 log_10_ pfu	i.v.	5	Viremic from day 1 up to day 4. One animal culled on day 3.
Morrill et al., 1989 [72]	10^5^ pfu	i.v.	17	All developed high viremia peaking at 48 h. *n* = 3 monkeys developed severe clinical signs: anorexia, cutaneous hemorrhage, epistaxis, and vomiting. One died on day 8 and two culled on days 6 and 15. Of the *n* = 14 which survived, 50% had a clinical illness (cutaneous rash, vomiting, or anorexia), and 7 had no evidence apart from brief pyrexia.
Morrill et al., 1989 [75]	5.0 log_10_ pfu	i.v.		Report from monkeys used above.
Cosgriff et al., 1989 [74]	5.0 log_10_ pfu	i.m. ^3^		Report based on the data from monkeys used above.
Morrill et al., 1990 [73]	10^5^ pfu	i.v.		Report based on the data from monkeys used above.
Morrill et al., 1991 [76]	10^5^ pfu	i.v.	3	All viremic with a peak after 24–48 h. One animal showed clinical signs characterised by petechia in the axillary and inguinal area alongside a temperature increase.
Morrill et al., 2003 [77]	5.0 log_10_ pfu	i.v.	4	All control monkeys challenged with virulent RVFV had detectable viremia for 3–5 days, and 75% (3/4) had poor appetite or anorexia, mild to moderate petechiation in the axillary and inguinal regions, and reduced activity from day 3 to 5.
Smith et al., 2011 [80]	7 log_10_ pfu	i.v.	4	All developed viremia, peaking on day 2. None presented with clinical illness.
Morrill et al., 2011 [78]	~10^5^ pfu	Aerosol	4	All showed elevated temperatures and viremia.
Morrill et al., 2011 [79]	3 × 10^6^ pfu	i.v.	3	Reduced activity during the first 2 days. Viremia started at 24 h and continued to day 3.
	~5 × 10^5^ pfu	Aerosol	5	All showed viremia on days 3 and 4. One monkey had a mild transient elevation in rectal temperature coinciding with the recovery of the virus from the oropharyngeal swab.
Hartman et al., 2014 [81]	5.04 or 5.67 log_10_ pfu	Aerosol	2	Biphasic fever, but no other clinical signs indicative of disease.
Bian et al., 2023 [82]	N/A	N/A	N/A	Immunogenicity study, no challenge.

^1^ i.p., intraperitoneal; i.c., intracranial; s.c., subcutaneous; i.n., intranasal; i.v, intravenous; s.c., subcutaneous. ^2^ Number of animals in untreated control groups. ^3^ Presumed error in the manuscript. ^4^ Animals with encephalitis received starch via the i.c. route alongside i.p. RVFV challenge. ^5^ MIPLD_50_, mouse intraperitoneal lethal dose of 50%.

**Table 2:** Overview of RVFV studies reported in cynomolgus macaques.

Publication	Challenge Dose	Challenge Route ^1^	Number	Outcomes
Findlay et al., 1936 [66]	Unknown	i.c.	1	Viremia and encephalitis, culled at 6 days post-challenge.
Easterday, 1965 [69]	Unknown	Unknown	Unknown	Viremia and febrile.
Hartman et al., 2014 [81]	5.04 and 5.67 log_10_ pfu	Aerosol	2	Biphasic fever, but no other clinical signs indicative of disease.

^1^ i.c., intracranial.

**Table 3:** Overview of RVFV studies reported in marmosets.

Publication	Challenge Dose	Challenge Route ^1^	Number ^2^	Outcomes
Findlay et al., 1932 [83]	Unknown	s.c.	3	Short febrile period followed by subnormal temperature drop. Two common marmosets recovered, but the other subspecies died; likely from non-RVFV causes.
Smith et al., 2011 [80]	7 log_10_ pfu	i.v.	4	All showed viremia apart from one that succumbed on day 2. Two others showed clinical illness including anorexia, decreased activity, and ruffled fur/hunched posture.
	7 log_10_ pfu	i.n.	4	A 100% mortality rate, with animals succumbing or being euthanised on days 8, 9, and 11. All showed neurological impairments.
	7 log_10_ pfu	s.c.	4	All showed viremia, and 50% succumbed or were euthanised. Three of four showed clinical illness similar to i.v. route.
	5 log_10_ pfu	s.c.	4	Same as with 7 log_10_ pfu, but with viremia levels significantly higher.
Hartman et al., 2014 [81]	Various (1.78–5.18 log_10_ pfu)	Aerosol	8	LD_50_ determined to be 3.5 × 10^3^ pfu. All exposed animals developed a biphasic fever. Clinical signs included dehydration and anorexia, as well as neurological signs late in the course of infection.
Smith et al., 2018 [85]	6.4 log_10_ pfu	s.c.	5	Viremia was detected in all animals, but none exhibited clinical signs of disease or experienced significant weight loss or temperature changes.
Schreur et al., 2022 [86]	10^7^ TCID_50_	s.c. + i.m.	4	One animal showed tremors starting on day 4 and substantial weight loss and was euthanised on day 15. The other animals did not show clinical signs.
	10^7^ TCID_50_	s.c.	6	Four animals showed clinical signs, such as unkempt coats and hunched postures. On days 13 and 14, the animals deteriorated to a lethargic state, reaching pre-defined humane endpoints.

^1^ i.v, intravenous; i.n., intranasal; s.c., subcutaneous. ^2^ Number of animals in untreated control groups.

**Table 4:** Challenge strains of RVFV used in NHP publications.

ZH501	ZH501 (Recombinant)	Other (Recombinant 74HB59 and 35/74)
Peters et al., 1986 [70]	Smith et al., 2011 [80]	Schreur et al., 2022 [86]
Peters et al., 1988 [71]	Hartman et al., 2014 ^2^ [81]	
Morrill et al., 1989 ^1^ [72]	Reed et al., 2014 ^2^ [92]	
Morrill et al., 1989 ^1^ [75]	Smith et al., 2018 [85]	
Cosgriff et al., 1989 ^1^ [74]		
Morrill et al., 1990 ^1^ [73]		
Morrill et al., 1991 [76]		
Morrill et al., 2003 [77]		
Morrill et al., 2011 [78]		
Morrill et al., 2011 [79]		

^1,2^ Same, or highly likely to be the same, cohort of animals reported.

**Table 5:** Genetic dissimilarity (% nucleotide sequence) between RVF strains.

% Nucleotide Changes			
	ZH501 ^1^ vs. 74HB59 ^2^	ZH501 vs. 35/74 ^3^	74HB59 vs. 35/74
L segment	0.020	0.038	0.034
M segment	0.024	0.051	0.047
S segment	0.022	0.037	0.028
NSs	0.026	0.047	0.034

Accession numbers: ^1^ DQ375406.1, ^2^ DQ375415.1, and ^3^ JF784386.1.

**Table 6:** Amino acid differences in the NSs protein region for RVF strains used in NHP challenge studies.

Strain	Amino Acid Position								
	23	75	133	167	202	217	239	242	250
ZH501	PHE	ALA	ASN	ALA	LYS	VAL	ILE	ILE	MET
74HB59	ILE	ALA	ASN	VAL	LYS	ALA	ILE	VAL	MET
35/74	ILE	VAL	SER	VAL	ARG	ALA	VAL	VAL	ILE

## Data Availability

No new data were created or analysed in this study. Data sharing is not applicable to this article.
